# Case Report: Functional MR imaging of alveolar soft part sarcoma

**DOI:** 10.3389/fonc.2025.1602577

**Published:** 2025-09-09

**Authors:** Jie Li, Zhaoyang Yang, Jiuming Jiang, Lihua Gong, Meng Li

**Affiliations:** ^1^ MRI Room of Qingdao Jiaozhou Central Hospital, Qingdao People’s Hospital Group (Jiaozhou), Jiaozhou, Shandong, China; ^2^ Department of Pathology, National Cancer Center/National Clinical Research Center for Cancer/Cancer Hospital, Chinese Academy of Medical Sciences and Peking Union Medical College, Beijing, China; ^3^ Department of Diagnostic Radiology, National Cancer Center/National Clinical Research Center for Cancer/Cancer Hospital, Chinese Academy of Medical Sciences and Peking Union Medical College, Beijing, China

**Keywords:** alveolar soft part sarcoma (ASPS), dynamic contrast-enhanced magnetic resonance imaging, intravoxel incoherent motion (IVIM), imaging, diagnosis

## Abstract

This study presents a rare case of acinar soft tissue sarcoma and provides a detailed analysis of its multi-parameter quantitative functional MRI (fMRI) characteristics. The patient, a 16-year-old male, was diagnosed with acinar soft tissue sarcoma after presenting with a progressively enlarging mass in the right thigh. To determine the extent and nature of the lesion, MRI examinations were performed using conventional plain scans, contrast-enhanced scans, dynamic contrast-enhanced magnetic resonance imaging (DCE-MRI), and intravoxel incoherent motion (IVIM) for quantitative analysis. Functional MRI imaging not only provides crucial diagnostic and differential diagnostic information for acinar soft tissue sarcoma, but also reveals through DCE-MRI and IVIM imaging indicators that ASPS exhibits high cellular density, increased vascular permeability, and abundant neovascularization. The local microcirculation characteristics offer vital insights into the tumor’s biological behavior and prognosis. Furthermore, functional MRI aids in precise diagnosis while providing references for surgical planning, postoperative adjuvant therapy, long-term follow-up evaluation, and recurrence risk prediction.

## Introduction

Alveolar soft part sarcoma (ASPS) is a rare soft tissue sarcoma and a malignant soft tissue tumor with indeterminate differentiation according to the 2020 version of the “WHO Classification of Soft Tissue and Bone Tumors” (1). ASPS is likely to occur in young and middle-aged populations, with a higher incidence in females than in males. The early symptoms and signs are not obvious. It often manifests as a painless mass, which is easy to ignore, and the first manifestation can be metastasis in other locations. MRI is the best imaging examination method for the detection and diagnosis of soft tissue tumors and the evaluation of treatment efficacy because it has high tissue resolution. On conventional MRI, ASPS is shown as an significant and persistent enhancement mass with visible tumor margins and/or internal thickening, dilation, tortuous blood vessels (2–4). With the advancement of imaging technology, MRI has also developed from conventional morphological imaging to quantitative functional imaging. For the first time, this study reported the multiparametric quantitative functional MRI features of the ASPS of the right thigh to increase awareness of the application of new imaging technologies in the ASPS.

## Case description

**Figure 1:**
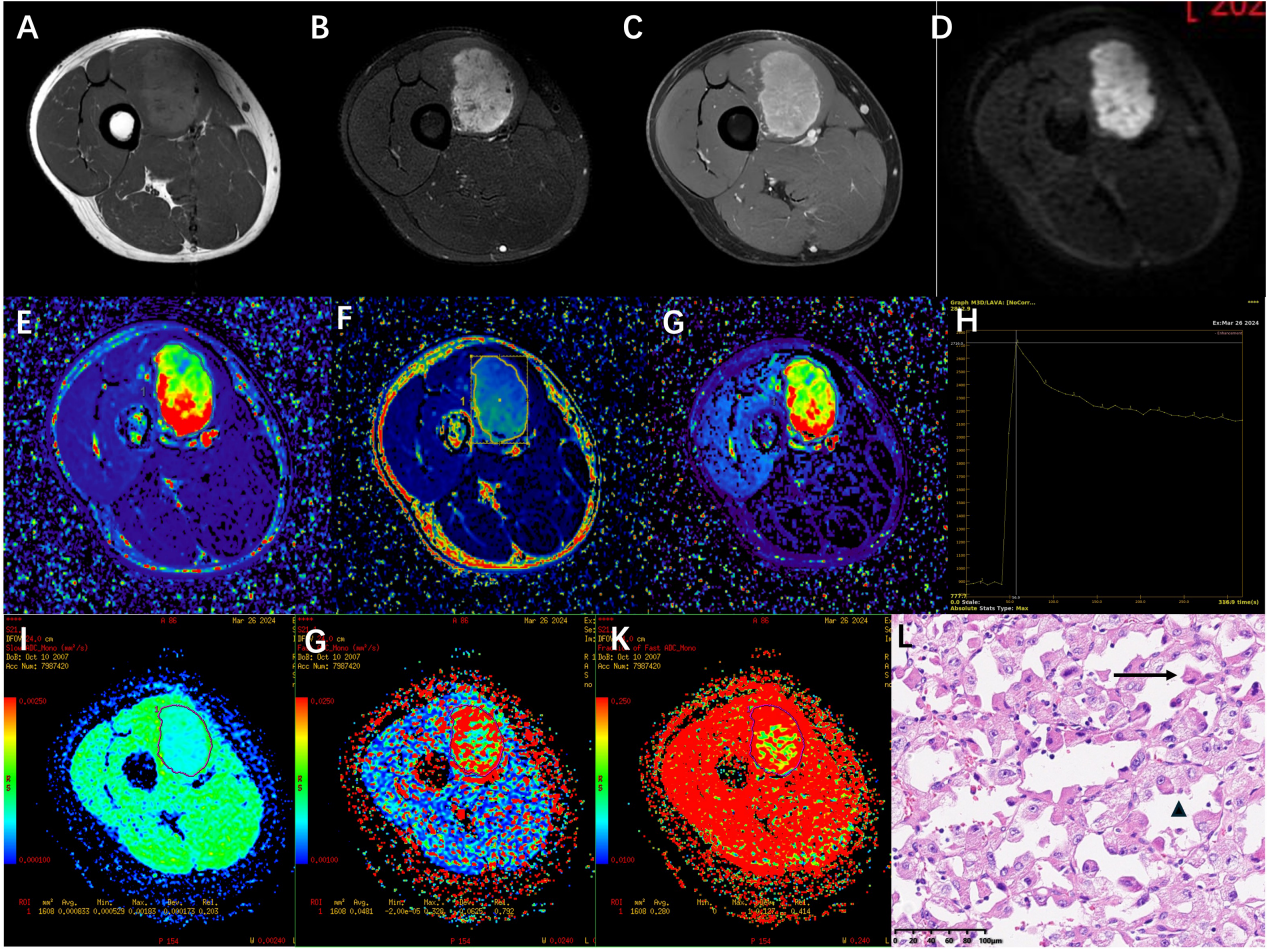
**(A-D)** a tumor of approximately 5.2cm×3.7cm×5.3 cm was observed in the vastus medialis muscle group of the right thigh. The signal was slightly high on T_1_WI, heterogeneously high on T_2_WI/FS in the fat-suppressed sequence, high on DWI, and obvious enhancement on the enhanced scan. **(E-G)** shows pseudocolor images of the DCE-MRI quantitative parameters K^trans^, K_ep_ and V_e_. The K^trans^, K_ep_ and V_e_ value of this soft tissue tumor ROI were (0.81 ± 0.58)/min, (3.19 ± 2.04)/min and 0.23 ± 0.07 respectively. **(H)** TIC curve the figure shows that the signal intensity during the baseline phase is low and stable.After the injection of contrast agent (approximately 24 seconds), the signal intensity rapidly increased and reached its peak (approximately 2716) at the 8th phase (approximately 56.9 seconds).It then gradually declined and stabilized, remaining at a relatively high level of approximately 2200 to 2300. **(I-K)** shows pseudocolour maps of the IVIM quantitative parameters D, D^*^, and f. The D, D^*^, and f value of this soft tissue tumor ROI were 0.83 ± 0.17×10 ^-3^ mm ^2^/s, 48.1 ± 6.25× 10 ^-3^ mm ^2^/s, and 28% respectively. **(L)** The pathological biopsy image shows that the tumor cells are arranged in acinar patterns with abundant blood sinuses (↑). The tumor cells have abundant cytoplasm, eosinophilic or hyaline vacuolar morphology (▴), and obvious nuclear atypia with prominent nucleoli (HE ×200).

A 16-year-old man was recently diagnosed with a tumor in his right thigh approximately 1 and a half years prior, which had increased significantly. To clarify the scope and nature of the lesion, an MRI examination was performed. Conventional plain and enhanced MRI scans revealed a tumor approximately 5.2 cm × 3.7 cm × 5.3 cm in the vastus medial muscle of the right thigh; the signal was iso- or slightly hyperintensive on T_1_WI; the fat-suppression sequence on T_2_WI (T_2_WI/FS) revealed a heterogeneous high signal, a high signal on DWI, obvious enhancement on the enhanced scan, and multiple tortuous and thickened vascular shadows in and around the lesions (**Figures 1A–D**). Dynamic contrast-enhanced magnetic resonance imaging (DCE-MRI) and intravoxel incoherent motion (IVIM) was performed and the sequence and parameters are shown in **Table 1**. Within the region of interest (ROI), the quantitative parameters derived from DCE-MRI were as follows: volume transfer constant (K^trans^), and extravascular extracellular volume fraction (V_e_), the rate constants (K_ep_) were 0.81 ± 0.58/min, 0.23 ± 0.07, and 3.19 ± 2.04/min, respectively (**Figures 1E–G**). In addition, the true diffusion coefficient (D) determined via IVIM, the pseudodiffusion coefficient (D^*^), and the perfusion fraction (f) were 0.83 ± 0.17×10^-3^ mm2/s, 48.1 ± 62.5 ^×10-3^ mm2/s, and 28%, respectively (**Figures 1I–K**).

**Table 1:** The parameters of DCE-MRI and IVIM scan.

Parameter	DCE-MRI	IVIM-DWI
Sequence type	3D T_1_-weighted spoiled GRE (LAVA)	Single-shot EPI
Plane	Axial	Axial
TR/TE_1_/TE_2_ (ms)	4.1/1.1/2.2	3000/65/–
Flip angle (°)	15	90
Slice thickness/gap (mm)	4.0/0	4.0/1
FOV (freq/phase) (mm)	300 × 240	250 × 250
Matrix	256 × 192	128 × 128
NEX	1	1–6
Bandwidth (Hz/pixel)	142.86	250
Frequency encoding direction	R/L	R/L
Number of phases	40	–
b-values (s/mm²)	–	0, 10, 20, 40, 60, 80, 100, 150, 200, 400, 800, 1000, 1200, 1500
Acquisition time	5 min 24 s	4 min 43 s

DCE, Dynamic contrast-enhanced magnetic resonance imaging; IVIM, intravoxel incoherent motion; GRE, gradient echo; LAVA, Liver Acquisition with Volume Acceleration; EPI, echo-planar imaging; NEX, number of excitations; FOV, field of view; TR, repetition time; TE, echo time; R/L, right/left.

**Figure 2:**
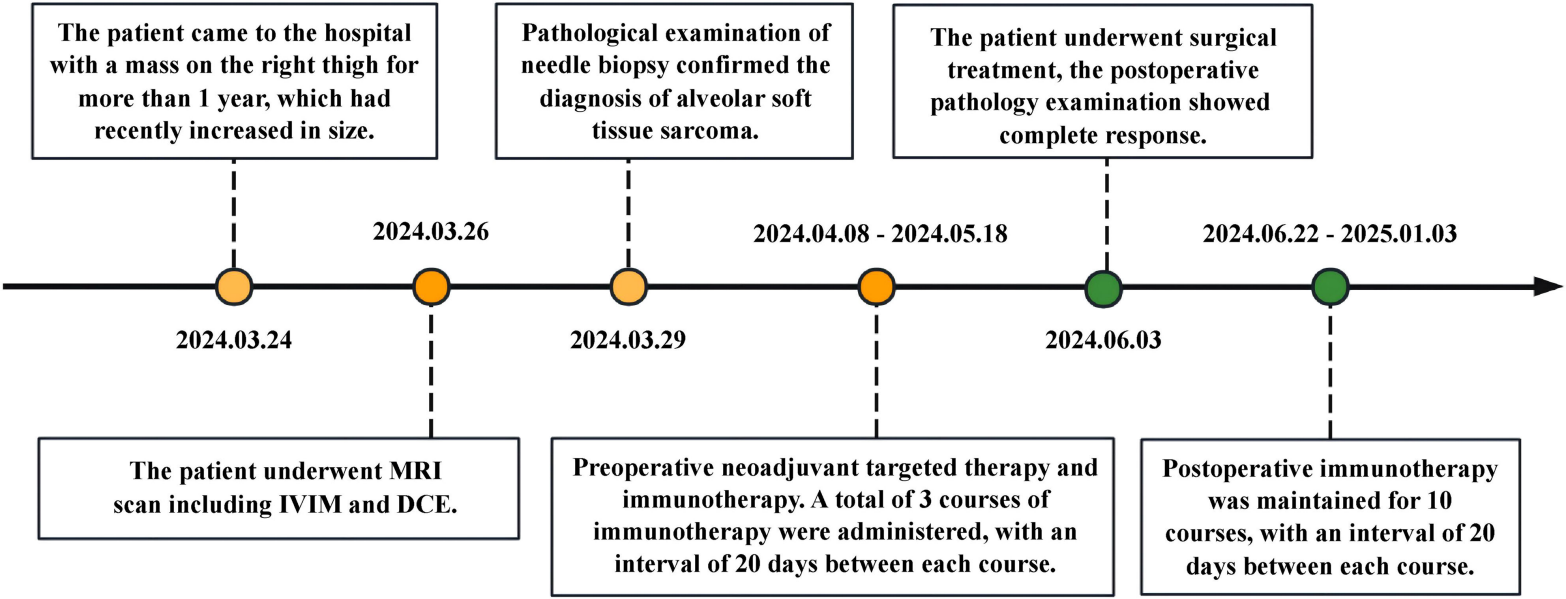
Timeline with relevant data about the onset, diagnosis, and therapy of the patient with alveolar soft part sarcoma.

The patient was admitted on March 24, 2025, and underwent comprehensive MRI and CT examinations on March 26. The integrated CT scan encompassing the chest, abdomen, and pelvis revealed no evidence of metastasis. Puncture on March 29, 2024, revealed tumor cells arranged in an acinar pattern with intervals rich in blood sinuses. The tumor cells exhibit copious cytoplasm, which may be eosinophilic, clear, or vacuolated, accompanied by striking nuclear pleomorphism and conspicuous nucleoli (HE ×200) (**Figure 1L**). Immunohistochemistry revealed: Vimentin (3+), TFE3 (3+), Ki-67 (5%+), S - 100 (-), confirming the pathological diagnosis of ASPS. Following a thorough assessment, the patient received treatment at another hospital, including three courses of preoperative neoadjuvant targeted therapy (Anlotinib, 12mg QD PO) combined with immunotherapy (Sintilimab, 200mg I.V.) between April 8, 2024 and May 18, 2024. MRI re-examination at external hospital demonstrated a substantial reduction in lesion size, heterogeneous internal signal characteristics, and a notable decrease in the number of blood vessels both within the lesion and along its margins. On June 3, 2024, the patient underwent surgical resection, and postoperative pathology confirmed significant treatment-related changes within the ASPS, with no detectable tumor cells remaining. Following surgery, the patient underwent a regimen of 10 maintenance immunotherapy courses at an external hospital, spanning from June 22, 2024, to January 3, 2025. The patient has been followed up to date, maintaining consistently stable vital signs and health status, while exhibiting no evidence of tumor relapse or distant metastasis. (**Figure 2**).

## Discussion

ASPS accounts for less than 1% of sarcoma incidence, predominantly occurs in young and middle-aged adults, and is most commonly found in the lower extremities. Clinically, the early symptoms of ASPS are not obvious, usually manifesting as a painless, gradually enlarging soft tissue mass. The onset age, location, and clinical characteristics of this case are consistent with those reported in the literature (5–7). In this case, the MRI routine examination and MRI contrast-enhanced scan findings of homo sapiens are consistent with previous imaging studies, Pathological findings indicate that the tumor cells are arranged in an acinar pattern, with fissure-like or sinusoidal capillary networks lined by a single layer of flattened endothelial cells between the acini, The pathological manifestations are also highly consistent with the imaging findings (8, 9). The above manifestations all indicate that the tumor has an extremely abundant blood supply. ASPS needs to be differentiated from other hypervascular soft tissue tumors such as aggressive fibromatosis, solitary fibrous tumor etc (10–12).

In recent years, multiparametric MRI—notably DCE-MRI and IVIM—has demonstrated considerable promise for the qualitative and quantitative assessment of soft tissue tumors (13). DCE-MRI parameters provide insight into the vascular permeability and hemodynamic characteristics of soft tissue tumors (14), not only distinguishing benign from malignant tumors but even identifying different malignant subtypes. Both K^trans^ and K_ep_ effectively distinguish between benign and malignant soft tissue tumors, while K_ep_ also effectively distinguishes high-proliferation and low-proliferation subtypes within malignant tumors (15). IVIM-MRI can more precisely characterize the tumor microenvironment status of via quantitative parameters (16, 17). IVIM leverages multi-b-value DWI to examine water molecule movement characteristics in tissues via a biexponential model, empowering multi-parameter quantitative analysis without requiring exogenous contrast agents. The D value captures the unrestricted diffusion motion of water molecules. This measure is inversely proportional to tissue cellular density, establishing it as a highly accurate diagnostic tool for differentiating benign from malignant soft tissue tumors, particularly for non-myxoid varieties (18, 19). Meanwhile, D* and f values reflect the diffusion state of water molecules within the microcirculation and the contribution of microperfusion, respectively, illuminating blood flow and capillary richness within the tumor.

This investigation employed functional MRI imaging techniques—specifically DCE-MRI and IVIM sequences—to conduct a thorough assessment of the tumor microenvironment and vascular functionality. By analyzing the TIC curve, this case reveals the tumor signal intensity rapidly ascends to its peak within a remarkably short time frame, characterized by a pronounced steep ascending slope and a relatively confined area under the peak. This pattern signifies the contrast agent rapidly arrives at the tumor region and accumulates within the tumor tissue. Meanwhile, the curve displays a more gradual descent, suggesting the contrast agent is excreted more slowly or exhibits retention properties. Research reveals that in neoplastic lesions, the elevation of the area under the curve and the rise slope, coupled with a shortened time to peak, correlate with enhanced regional blood perfusion and/or heightened vascular permeability. These changes may reflect intensified tissue cellular function (20). The TIC curve for this patient exhibits features that align with these observations. Quantitative analysis demonstrated that the elevated K^trans^ value indicates significantly heightened vascular permeability and immature vasculature within the tumor region. A substantial standard deviation signifies considerable variability in K^trans^ value distribution, suggesting potential permeability heterogeneity within the lesion. A lower V_e_ value signifies that tumor cells are more densely packed within the tumor tissue, suggesting that ASPA is a tumor with relatively dense cellularity. In this instance, the patient displayed elevated K^trans^ and K_ep_ values. The underlying cause likely stems from the tumor’s intensified need for oxygen and nutrients, requiring the formation of more immature, highly permeable neovascularization to deliver nourishment. This enables accelerated blood flow into the hemorrhage circulation, ultimately resulting in increased K^trans^ values, and rapidly directs blood flow into the blood vessels, ultimately leading to an increase in K_ep_. This suggests that ASPS exhibits greater local microcirculatory vascular permeability, with enlarged gaps between vascular endothelial cells or poorer vascular maturity in the affected area. DCE-MRI pseudocolor images provide an intuitive visualization of the hemodynamic characteristics within ASPS tumors. IVIM imaging of this case indicates that the lower D value in ASPS may be related to the higher cellular density of ASPA, where densely packed cells restrict water molecule diffusion. D* represents the diffusion state of water molecules in the microcirculatory capillary network. A higher D* value indicates increased intratumoral blood flow velocity and abundant blood supply in ASPA. The f-value quantifies the volumetric proportion of microcirculatory water molecule diffusion relative to the total diffusion effect, serving as a key indicator of tissue capillary density (21). An elevated f-value corresponds to heightened blood perfusion within ASPS tissues, signifying that microcirculation contributes a substantially greater fraction to the observed signal. The decrease in ADC and D values of ASPA may be attributed to the high density of tumor cells, where the extracellular space is smaller than that of normal tissue, resulting in reduced diffusion of free water molecules. Higher D* and f values both reflect the microperfusion status of capillaries in ASPS tissues, indicating increased neovascularization and enhanced blood flow perfusion within the tumor. IVIM pseudo-color images offer a more intuitive visualization of intricate micro-environmental features within ASPS.

By integrating DCE-MRI and IVIM imaging parameters, ASPS exhibits high cellular density, elevated vascular permeability, and abundant neovascularization. The characteristics of local microcirculation provide crucial information for the biological behavior and prognosis of tumors. Functional MRI not only aids in precise diagnosis, but also provides reference for surgical planning, postoperative adjuvant therapy, and long-term follow-up evaluation.

Although surgery is still the cornerstone of radical treatment for soft tissue tumors, in recent years, neoadjuvant therapy has been increasingly used in high-grade and deep soft tissues patients (22). As it can improve the tumor microenvironment, shrunk the tumor, facilitate surgery, improve the surgical effect. At the 2023 American Society of Clinical Oncology (ASCO) Annual Meeting, multiple research studies reported new advancements in immunotherapy and targeted therapy for soft tissue sarcoma (23, 24). In this case, the patient received neoadjuvant targeted therapy and immunotherapy, with the preoperative tumor showing significant reduction in size compared to the primary lesion. Postoperative pathology indicated tumor complete remission. Follow-up to date shows good condition with no signs of relapse or distant metastasis.

In summary, as a rare malignant soft tissue tumor, ASPS is characterized by high cellular density, elevated vascular permeability, and abundant microcirculation. Functional MRI imaging can comprehensively reflect the tumor microenvironment and vascular function information, providing new imaging evidence for tumor diagnosis, surgical planning, postoperative adjuvant therapy, and long-term efficacy evaluation. Moreover, the local microcirculation status of the tumor is closely related to the risk of relapse, and accurate assessment of this indicator is of great significance for the comprehensive management of soft tissue sarcoma.

## Data Availability

The original contributions presented in the study are included in the article/supplementary material. Further inquiries can be directed to the corresponding authors.
